# Advances in the isolation, cultivation, and identification of gut microbes

**DOI:** 10.1186/s40779-024-00534-7

**Published:** 2024-06-03

**Authors:** Meng-Qi Xu, Fei Pan, Li-Hua Peng, Yun-Sheng Yang

**Affiliations:** 1 Department of Gastroenterology and Hepatology, the First Medical Center of Chinese, PLA General Hospital, Beijing, 100853 China; 2 Medical School of Chinese PLA, Beijing, 100853 China

**Keywords:** Gut microbes, Culturomics, Microbial identification, Droplet microfluidics, Strain-level investigation

## Abstract

The gut microbiome is closely associated with human health and the development of diseases. Isolating, characterizing, and identifying gut microbes are crucial for research on the gut microbiome and essential for advancing our understanding and utilization of it. Although culture-independent approaches have been developed, a pure culture is required for in-depth analysis of disease mechanisms and the development of biotherapy strategies. Currently, microbiome research faces the challenge of expanding the existing database of culturable gut microbiota and rapidly isolating target microorganisms. This review examines the advancements in gut microbe isolation and cultivation techniques, such as culturomics, droplet microfluidics, phenotypic and genomics selection, and membrane diffusion. Furthermore, we evaluate the progress made in technology for identifying gut microbes considering both non-targeted and targeted strategies. The focus of future research in gut microbial culturomics is expected to be on high-throughput, automation, and integration. Advancements in this field may facilitate strain-level investigation into the mechanisms underlying diseases related to gut microbiota.

## Background

The human gut harbors approximately 4 × 10^13^ microbes, a number comparable to the total count of human cells [1]. Recent advancements in multi-omics technologies and cost reductions in testing have significantly contributed to our understanding of the composition and function of the gut microbiome. More evidence indicates a strong correlation between the gut microbiome and human health, with conditions such as inflammatory bowel disease, colorectal cancer (CRC), metabolic syndrome, and autism spectrum disorder intricately linked to the gut health status [2–10].

Culture-independent approaches have played a crucial role in advancing our understanding of the gut microbiota. The Unified Human Gastrointestinal Genome (UHGG) collection contains 204,938 non-redundant genomes from 4644 prokaryotic species inhabiting the gut [11], whereas the Human Reference Gut Microbiome has expanded to 5414 distinct species [12]. However, more than 70% of the UHGG species lack a cultured representative [11]. These unknown ‘dark matter’ significantly hinder our comprehension of gut microbe functionalities and their interactions with the host. Recently, several large-scale cultivation initiatives have been undertaken [13–20], leading to the successful culturing of over 1500 microbial species found in the gut [20]. Although this progress is exciting, it remains insufficient as our primary challenge lies in expanding the culturable repertoire of gut microbiota and constructing an overall reference genome database.

The wealth of data generated by multi-omics studies is poised to drive advancements in gut microbiome research and precision medicine [21–26]. Although multi-omics research can reveal the correlation between disease and gut microbiota, as well as provide insights into the microbial and host interaction network, subsequent validation experiments remain imperative. To explore the precise role of the gut microbiome in disease progression [27], design synthetic microbial communities [28], and ultimately translate the gut microbiome research into clinical applications, it is essential to initially obtain viable gut microbes. Acquiring target microorganisms from the vast array of intestinal microbes for in vitro culture poses a technical challenge encountered in microbiome research.

**Fig. 1:**
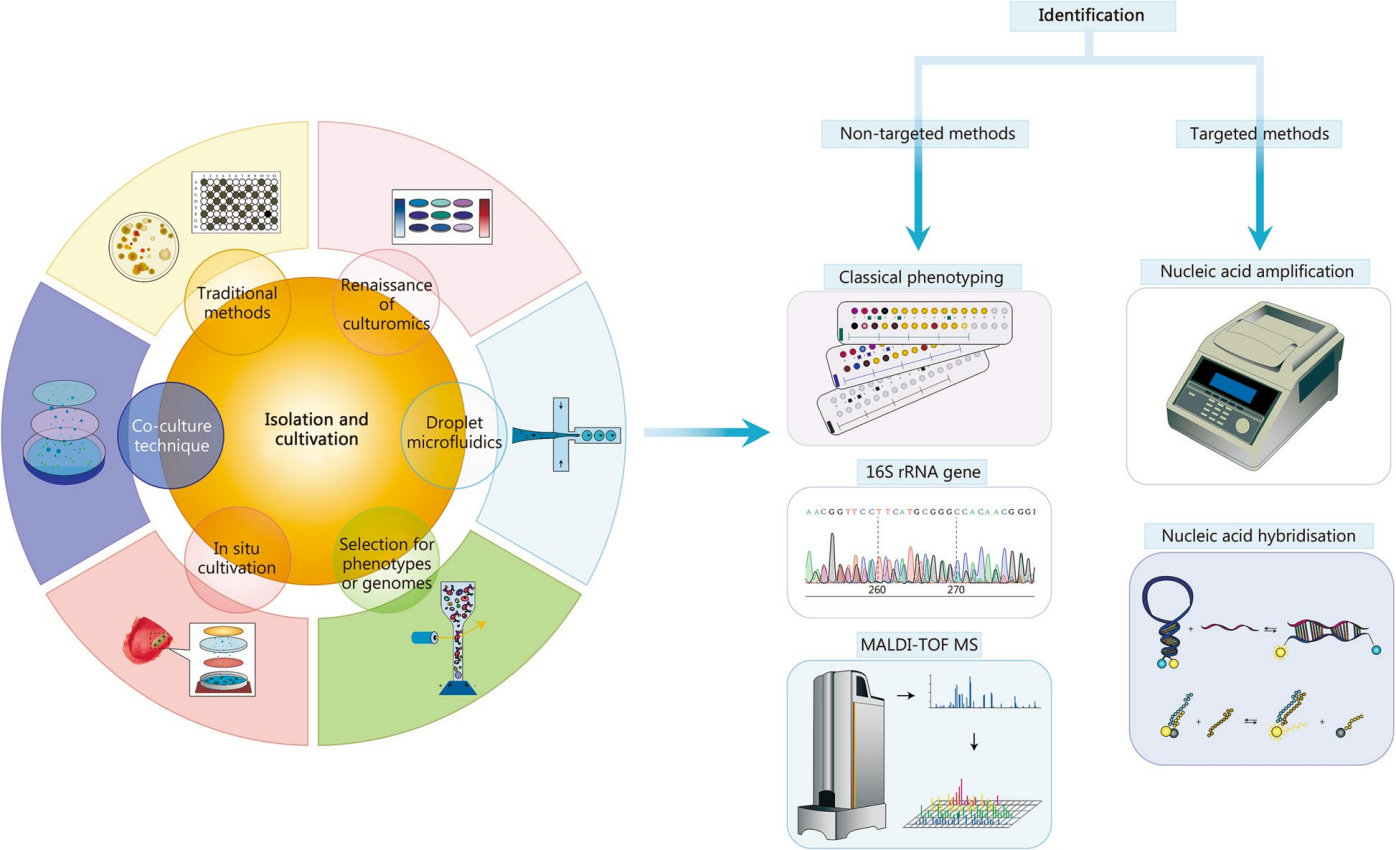
Advances in the isolation, cultivation, and identification of gut microbes. The isolation and cultivation techniques of gut microbes are depicted on the left. Traditional methods involve plate-based techniques and limiting dilution in liquid media. The renaissance of culturomics focuses on refining and improving culture media and conditions to enhance species diversity through streamlined procedures. Droplet microfluidics in the cultivation of gut microbes is characterized by high-throughput, automation, single-cell, and miniaturization, which are compatible with anaerobic workstations. Phenotypic and genomics selection mostly rely on FACS. In situ cultivation and co-culture techniques are based on membrane diffusion. The identification technique used for gut microbes is depicted on the right. Non-targeted identification methods include classical phenotyping, 16S rRNA gene sequencing, and mass-assisted laser desorption/ionization time-of-flight mass spectrometry (MALDI-TOF MS). Targeted identification mainly employs the principle of nucleic acid amplification or hybridization techniques

Gut microbiome research is evolving from mere description and investigation to in-depth studies of mechanisms and potential clinical therapies. This shift presents new demands and challenges for research methods and techniques. In this review, we delve into the latest advancements in gut microbe culturomics, specifically focusing on the isolation, cultivation, and identification of gut microbes, to offer methodological support for a comprehensive study of the gut microbiome (Fig. 1) [13, 14, 29–53].

## Gut microbial isolation and cultivation

### Traditional methods

The conventional method for isolating and culturing microbes primarily relies on plate-based techniques such as spread-plating and streak-plating procedures. Under appropriate conditions, colonies can develop on solid media and are visible to the naked eye without magnification. In practice, direct microscopic count yields a higher number of microbes in natural samples compared to plate count, which is known as the great plate count anomaly phenomenon [54]. This difference may be attributed to the fact that the media does not accurately represent the real natural environment, resulting in most microorganisms being unable to thrive. Other factors include symbiosis between cells or viable but non-colony-forming cells.

As some microbes cannot form colonies, limiting dilution in liquid media serves as another effective method for isolating individual microbial cells. Furthermore, liquid media offer a simpler experimental process and higher throughput. Goodman et al. [29] initially designed the Gut Microbiota Medium and then performed limiting dilution in 384-well microplates to achieve single-cell isolation followed by liquid anaerobic cultures. From fecal samples from a single donor, they obtained a total of 1172 isolates belonging to 48 identified species and other previously cultured species.

It is worth noting that most gut microbiota are anaerobic organisms requiring complex media with various supplements and specialized equipment to provide an anaerobic environment. The Hungate anaerobic roll-tube technique is a traditional approach for cultivating anaerobic microbes [55]. Additionally, chemically generated anaerobic systems such as AnaeroPack [56] and BBL GasPak [57] are commonly used, particularly in smaller labs. The commercialization of anaerobic chambers has also led to the development of customized systems that integrate automated colony operation workstations, albeit at a significant cost [58, 59]. These systems are complex to operate and require professional training, undoubtedly increasing the difficulty involved in culturing gut microbiota.

### The renaissance of culturomics

Culturomics employs a variety of culture conditions to facilitate the growth of fastidious bacteria, specifically utilizing high-throughput culture approaches combined with advanced techniques such as 16S rRNA gene sequencing and mass-assisted laser desorption/ionization time-of-flight mass spectrometry (MALDI-TOF MS) for bacterial identification. In 2012, Lagier et al. [13] conducted a groundbreaking study on the culturomics of gut microbes. They collected fecal samples from 3 volunteers and employed 212 different culture conditions, resulting in over 30,000 isolates from 314 species, half of which were newly discovered in the human gut. Based on their findings, 70 effective culture conditions were identified, with 18 being optimal for the isolation and cultivation of microbes from fecal samples. Additionally, blood culture bottles, rumen fluid, and sheep blood were confirmed as critical nutrient substrates for microbial growth. Subsequently, Lagier et al. [14] conducted large-scale culture studies that expanded the number of known human gut microbiota to include 1525 species. Diakite et al. [30] further investigated the optimization and standardization of the culturomics workflow by confirming 16 specific culture conditions that covered 98% of isolates from previous work while simplifying the process without compromising microbial diversity. Chang et al. [31] discovered that extending pre-incubation periods, introducing fresh medium into blood culture bottles, and determining appropriate sampling frequency effectively reduced labor consumption while enhancing culture yield.

Overall, culturomics emphasizes the continuous refinement and enhancement of culture media and conditions to optimize species diversity through streamlined procedures [32, 33]. One advantage of using solid media is its ability to facilitate visual identification and selection of microbiota based on their observable morphologies. However, this method remains associated with fundamental and repetitive labor-intensive processes, including plate pouring, spread plating, and colony picking. Despite the development and commercialization of various automated equipment such as automatic media streakers and automatic microbiota selectors, their high costs and compatibility challenges with anaerobic platforms have limited their application in gut microbiota research. Therefore, there is a need for the development of novel technical solutions.

### Droplet microfluidics

The utilization of droplet-based microfluidic systems enables precise manipulation of discrete fluid volumes containing immiscible phases, thereby facilitating accurate control over flow patterns and lengths for the production of water-in-oil or oil-in-water microdroplets [60]. Due to their dispersion, each microdroplet can establish an enclosed microreaction environment. The introduction of microscale droplet generation has revolutionized microbial isolation and cultivation by offering high-throughput capabilities, automation, single-cell analysis, and miniaturization features inherent in droplet microfluidics.

#### SlipChip

Du et al. [34] developed a SlipChip device for the generation of nanolitre-scale droplets, which were subsequently manipulated through sliding, one for microbial cultivation and the other for destructive testing. When combined with polymerase chain reaction (PCR), this approach enables the targeted isolation and cultivation of *Bacteroides vulgatus*, a common gut resident [35, 36]. SlipChip represents a significant endeavor in applying droplet microfluidics to gut microbes, facilitating automated as well as high-throughput isolation and cultivation of gut microbes. However, the sliding operation limits the throughput of droplet generation, manipulating only hundreds of microbial cells in a single assay. Although there has been an improvement in throughput compared to traditional methods, meeting the requirements for isolating and culturing rare species from thousands of gut microbes and conducting large-scale intervention experiments remains unmet.

#### End-to-end droplet microfluidic system

Watterson et al. [37] designed an end-to-end droplet microfluidic system integrated with an anaerobic incubator, enabling the simultaneous production of millions of picolitre single-cell droplets. Due to Poisson statistics commonly used in passive cell capture processes [61], only a fraction of the resulting droplets can effectively contain a single cell when there are background populations of empty droplets. Watterson et al. [37] also devised an image-based sorting algorithm and microfluidic control system for sorting bacterial colonies in droplets based on colony density, eliminating the need for fluorescent strains or reporters. Traditional plate-based methods favor the cultivation of fast-growing microbiota while posing limitations for slow-growing ones. Furthermore, some microbiota may be inhibited by competing interactions within their environment. The physical separation provided by droplet microfluidics effectively addresses these issues, leading to a significant increase in microbiota diversity and low-abundance microbial species. More importantly, isolation, cultivation, and sorting are tightly integrated into a standard anaerobic glove box. However, manipulation of picolitre-sized droplets poses challenges as the sorted droplets are pooled and gathered collectively rather than individually. Hence, further separation, purification, and scale-up remain necessary for subsequent studies.

#### Microfluidic streak plate

In a separate study, Jiang et al. [38] presented a straightforward method to directly index each droplet and achieve the addressability necessary for precise manipulation of droplets without subsequent re-separation. They introduced the microfluidic streak plate, which is based on the conventional streak plate technique. This device can generate nanolitre droplets initially and then arrange them in spiral arrays in Petri dishes pre-filled with carrier oil for individual cell cultivation purposes. Targeted recovery was achieved by imaging and aligning with the assistance of a stereoscope, followed by manual selection using sterile toothpicks. Furthermore, integrating the microfluidic streak plate platform with an anaerobic incubator to termite gut microbe culture has led to the identification of a potential new taxon [62].

#### Single-cell microlitre-droplet screening system

The droplets in the aforementioned studies range from picolitre to nanolitre in size, necessitating the use of a microscope for their manipulation [34–38, 62]. Some of these droplets require re-separated after enrichment or manual sliding or picking [34, 38]. Jian et al. [39] introduced the single-cell microlitre-droplet screening system (MISS Cell), which is capable of generating 2.0 to 2.5 µl droplets with a throughput of 10^4^ cells. MISS Cell consists of 4 interconnected modules: the sampling module, microfluidic chip module, droplet storage and culture module, and droplet detection and collection module. The system employs spectral detection and specific algorithms for accurate identification and automated sorting of droplets. It also incorporates a unique mechanical structure that enables the automatic collection of individual droplets by docking with a standardized container through droplet printing. Compared to picolitre droplets, MISS Cell utilizes a simplified optical signal detection system that accurately identifies and precisely addresses the droplets, significantly enhancing the colony picking efficiency of high-throughput applications.

The features of the current typical high-throughput droplet microfluidic culture system for gut microbes are summarised in Table 1. Droplet microfluidics is characterized by its small size (ranging from picolitres to microlitres), independent nature of the droplets, high culture throughput, low reagent consumption, and compartmentalization cultivation. This makes it a significant advancement in gut culturomics studies. It enables the discovery of rare bacteria and the reconstruction of intestinal microbial diversity due to its exceptional cultural performance. Additionally, the compatibility of droplet microfluidic equipment with anaerobic devices greatly expands its application in the field of intestinal microbiology. However, there are certain limitations associated with this technology. According to the principles of Poisson distribution, the generation of single-cell encapsulated droplets is expected to result in a significant proportion of empty droplets [61]. The fundamental challenge lies in identifying and categorizing ‘microbe growing’ droplets and empty ones. Additionally, current methods for sorting droplets lead to their aggregation; thus, making it difficult to establish a one-to-one index for subsequent analysis. This poses a challenge in achieving an optimal balance between droplet throughput and addressability. Moreover, the process of isolation impedes the proliferation of organisms that rely on other microbial or host cells [63]. Hence, the co-encapsulation of two cross-feeding auxotrophic strains within a singular droplet may prove to be a viable and efficient strategy, which can also be utilized for studying the interactions between microbiotas [64].

**Table 1:** Characteristics of the current typical high-throughput droplet microfluidic culture system for gut microbes

Microfluidic platform	Droplet size	Throughput	Culture performance	Detecting and sorting method	Picking operation	Is it addressable?
Slipchip [34, 35]	nl	10^3^	Droplet culture in microcompartments of ‘replica-SlipChip’	Imaging with a microscope and no sorting	Picking manually with Eppendorf pipettors	Yes
End-to-end droplet microfluidic system [37]	pl	10^7^	Droplet culture in stacks	Imaging analysis using a high-frame rate camera and custom LabVIEW code, sorting via optical detection based on colony density	-	No
Microfluidic streak plate [38]	pl-nl	10^4^	Droplet culture in spiral arrays on petri dishes	Imaging and aligning with a stereoscope and no sorting	Picking manually with sterile toothpicks	Yes
Single-cell microliter-droplet screening system [39]	µl	10^4^	Droplet culture in sequence in Teflon tubes	Imaging analysis using a spectrometer and sorting via optical density	Automatic collection to standard container	Yes

In recent years, droplet-based microfluidic technology for single-cell isolation and sequencing has gained significant traction, enabling the cultivation of bacteria and even genome amplification within individual encapsulated cells [65]. Meng et al. [66] have employed the DREM cell platform to investigate microorganisms in the honeybee gut, facilitating strain-level analysis as well as the identification of potential novel species and specific functional strains. Additionally, the Microbe-seq technique developed by Zheng et al. [67] has been applied to analyze human fecal samples, providing species information at a strain-level resolution and facilitating in-depth functional research. Notably, this review article primarily focuses on the indexing, acquisition, and amplification of individual droplets for further causal research. These two research methods are essentially complementary.

### Selection for phenotypes or genomes

Current high-throughput isolation and cultivation methods aim to enhance our understanding of the microbial ‘dark matter’. Furthermore, selectively isolating organisms with specific functional characteristics or those belonging to particular taxonomic groups is another strategy for delving deeper into gut microbiome research.

One effective method involves the use of selective media, such as the *Bacteroides fragilis* (*B. fragilis*) bile-esculin agar, which has been specifically formulated to target the *B. fragilis* group [68]. Oberhardt et al. [69] constructed a web-based platform that predicts suitable media based on 16S rRNA gene sequences, thereby facilitating future cultivation efforts. By employing alcohol pre-treatment on stool samples, Browne et al. [15] targeted the sporulation phenotype, and successfully isolated spore-forming bacteria, leading to the discovery of 45 potential novel species.

The technique of fluorescence-activated cell sorting (FACS) separates a heterogeneous population of cells into multiple containers based on their distinct light scattering and fluorescent properties [70]. Various strategies employing fluorescent labeling have been developed to utilize FACS for the categorization of gut microbiota with specific phenotypes or genomes. Cross et al. [40] employed single-cell genomic data to identify genes encoding membrane-associated proteins, which were then used to generate engineered antibodies. Human oral samples were incubated with these fluorescently labeled antibodies and subsequently analyzed using flow cytometry. This reverse genomics approach facilitated the isolation and cultivation of three distinct species-level lineages of human oral Saccharibacteria (TM7) and SR1 bacteria, the latter belonging to a candidate phylum without any previously cultured representatives. Moreover, Batani et al. [41] optimized the conditions affecting cell viability during fluorescence in situ hybridization (FISH), combining it with FACS to selectively isolate and cultivate specific bacterial taxa based solely on their 16S rRNA gene sequences. Although these innovative methods can target specific phenotypic or genomic traits, the sample preparation process may result in reduced cell viability, while the equipment does not provide an anaerobic environment, posing challenges in recovering obligate anaerobes. Moreover, further isolation and cultivation are still required for the sorted bacteria.

### Membrane diffusion-based cultivation

#### In situ* cultivation*

Kaeberlein et al. [42] developed a diffusion chamber with 0.03 µm filter membranes on both sides, in which they placed environmental samples inoculated on an agar matrix. The equipment was placed in a natural setting to restrict microbial mobility, facilitating the natural exchange of environmental substances and eliminating the need for sophisticated medium design, thus enabling in situ microbe culture. Similarly, Gavrish et al. [43] developed a microbial trap where the agar matrix within the chamber lacked microbial inoculation. A 0.03 µm filter membrane was coated on the top of the chamber to prevent air pollutants, whereas a 0.2 mm filter membrane was placed at the bottom to allow entry of environmentally specific microbes and facilitate the natural interchange of environmental substances. Building upon this concept, Sizova et al. [44] designed a minitrap consisting of multiple micro-chambers made from a human oral prosthesis that can be worn by patients for 48 h to enable inoculation with oral anaerobic/aerobic microbes. This device revolutionized in situ isolation and cultivation of human oral microbes and offers significant advantages over conventional solid culture media in terms of microbiota quantity and species diversity.

The main concern for achieving isolation and cultivation of microorganisms lies in addressing their nutritional needs and providing optimal conditions. In the case of gut microbiota, it is both intuitive and effective to supplement with rumen fluid or sterilized fecal extract [71] and create an anaerobic environment to restore the intestinal state of the gut as closely as possible. In situ cultivation is a method that effectively mimics the natural growth conditions of microbes, thus eliminating the need for complex media design. However, its applications in studying gut microbiota are constrained by challenges such as device design and pick-and-place methodologies, highlighting the necessity for further technological advancements.

#### Co-culture technique

The growth of specific gut microbes depends on the metabolic support from other bacterial species. Tanaka et al. [45] designed a co-culture system using a soft agar layer composed of a basal 1.5% agar medium and two layers of 0.4% soft agar medium separated by a 0.22 µm filter membrane. This system successfully isolated and cultured 3 previously uncultured strains from fecal samples by enabling communication between microbes via the diffusion of soluble substances. Soft agar media allows for more rapid molecule diffusion compared to conventional 1.5% agar media, thereby promoting optimal growth conditions.

## Gut microbial identification

The development of culture techniques is inherently intertwined with the effectiveness of identification techniques. Currently, there are two main strategies employed for gut microbial isolation and cultivation: high-throughput methods aimed at reducing the number of uncultured microbes and restoring diversity, and targeted isolation approaches designed to facilitate in-depth research on specific microbes. Non-targeted and targeted identification strategies and technologies exhibit differences.

### Non-targeted identification

#### Classical phenotyping

The traditional method of microbial identification relies on phenotyping, which involves assessing various characteristics such as size, morphology, enzyme metabolism, carbon source utilization, organic metabolites, cellular fatty acid components, and other physiological and biochemical traits. Several commercial kits or automated systems are available for microbial phenotyping, with testing times ranging from 4 to 72 h [46]. However, classical phenotyping is not only labor-intensive but also lacks universal applicability.

#### 16S rRNA gene

Nucleic acid sequencing of the bacterial 16S rRNA gene has been extensively used for decades to identify clinical and environmental isolates and establish phylogenetic relationships. Due to its stability throughout biological evolution, the 16S rRNA, which is commonly found in prokaryotes and exhibiting is often considered a reliable indicator of biological evolution. The 16S rRNA gene contains sequences that span highly conserved, variable, and hypervariable regions, making it well-suited for examining genetic relationships among organisms with varying evolutionary distances [47].

Initially, pure culture identification relied on PCR amplification of the full-length 16S rRNA gene followed by Sanger sequencing, whereas next-generation sequencing has predominantly been used to analyze complex microbial communities. Characterizing numerous isolates from culturomics represents a significant and costly endeavor. Zhang et al. [48] reported a high-throughput identification method based on next-generation sequencing. They introduced two-sided barcodes during library preparation targeting bacterial 16S rRNA genes to label the plates and wells containing pure DNA templates of clonal cultures in 96-well plates. These samples were subsequently pooled for Illumina sequencing. Bioinformatic analyses were performed to identify cultures and ensure the traceability of each well according to the barcodes. This method enables simultaneous identification of microbes in 48 × 96-well plates, leading to substantial cost reduction and improved throughput.

#### MALDI-TOF MS

MALDI-TOF MS has been widely utilized as a valuable analytical chemistry tool for detecting large and small molecules. Employing specific algorithms to compare protein spectra with reference databases, enables accurate identification of microbes. In 2009, the initial application of MALDI-TOF MS in a routine clinical microbiology laboratory successfully achieved precise identification at both genus and species levels [49]. The remarkable speed at which MALDI-TOF MS operates allows for microbial identification within minutes. Due to its rapidity, cost-effectiveness, high-throughput, and minimal training requirement, MALDI-TOF MS has emerged as a globally standardized mainstream technology [50]. This advancement in MALDI-TOF MS has sparked a renaissance in culturomics by transforming microbial isolation and cultivation into a high-throughput process.

However, the effectiveness of utilizing MALDI-TOF MS for microbial identification heavily relies on the quality and quantity of database spectra available. The success rate and accuracy of identification are directly influenced by these factors. Currently, accessible public databases are limited and expensive, highlighting an urgent need for a comprehensive, high-quality mass spectrometry library specifically tailored towards gut anaerobic microbes.

### Targeted identification

#### Nucleic acid amplification

PCR is a widely used method for targeted identification of microbial nucleic acids, in which specific primers are designed for the target sequence and qualitative detection is achieved through exponential amplification via thermal cycling [72]. However, the integration of other equipment to achieve online monitoring becomes challenging due to the requirement of a precise temperature control module in the PCR thermal cycle reaction.

Isothermal amplification of nucleic acids is a straightforward process that rapidly and efficiently accumulates nucleic acid sequences at a constant temperature [73]. For instance, loop-mediated isothermal amplification (LAMP), an extensively studied isothermal technique, depends on primer sets with 4 to 6 specific primers for the identification of various regions in the target sequence. LAMP also provides uncomplicated qualitative detection through turbidity, colorimetry, and fluorescence [74]. Currently, commercially available *Mycobacterium tuberculosis* LAMP detection kits are widely used in diverse clinical diagnoses and treatments [75]. Due to its simple reaction conditions, LAMP can be well integrated with droplet microfluidic equipment and offers a wide range of applications in the identification and detection of microorganisms [51, 52].

#### Nucleic acid hybridization

Nucleic acid hybridization is a valuable tool for the identification of target genes or organisms. In FISH, fluorescently labeled oligonucleotides (typically 15 – 20 base pairs long), are specifically designed to bind to rRNAs within intact cells, thereby resulting in cellular fluorescence. Numerous adaptations of the technique have been documented, such as fixation-free FISH [76], in-solution FISH [77], and live FISH [41]. These methods can also be integrated with FACS for sorting labeled cells. However, one limitation of FISH is the need to remove unreacted probes through additional operational procedures, which restricts its potential for integrated applications.

Molecular beacons (MBs) are innovative nucleic acid probes consisting of 3 components: a loop region, a stem region, and a fluorophore/quencher. In their unbound state, MB adopts a hairpin conformation with the fluorophore and quencher nearby, resulting in fluorescence quenching. Upon binding to the complementary sequence, the conformation of MB changes, separating the fluorophore and quencher, leading to fluorescence emission that indicates the presence of the target sequence. Similarly, researchers have optimized the MBs and developed double-stranded molecular probes which are inherently compatible with liquid-phase hybridization detection without requiring probe elution. This considerably streamlines the experimental process [78]. Kaushik et al. [53] developed a DropDx platform by employing droplet microfluidics combined with fluorogenic hybridization probes for pathogen identification and antimicrobial susceptibility testing. Bacteria in urine samples are encapsulated into picolitre-sized droplets along with 16S rRNA‐specific peptide nucleic acid probes before undergoing on‐chip culture for 10 min followed by probe hybridization for 16 min. The fluorescence emitted by these droplets is then used to detect specific 16S rRNA sequences for identifying uropathogenic bacteria. Additionally, an antibiotic is introduced to perform antimicrobial susceptibility testing. The microfluidic platform integrates three modules: single-cell pathogen capture, sample preparation-free molecular probe-based detection, and nucleic acid amplification, as well as high-throughput quantitative determination through fluorescence analysis, thus enabling rapid, automated, and high-throughput clinical sample testing.

## Application and future of gut culturomics

### Culturomics and metagenomics are complementary to each other

The annotation of species in high-throughput sequencing necessitates the utilization of known species data through blasting [11]. The isolation, cultivation, and identification of gut microbes can expand the database of culturable gut microbes and promote genomics research. Li et al. [79] employed a combination of in-depth metagenomic sequencing and large-scale culturomics to reveal the distinctive structure of gut microbial communities within a Chinese longevity population. Only 42.17% of the isolated species were also detected using metagenomics, indicating clear complementarity between these two approaches. Certain microorganisms, despite their abundance in metagenomic analysis, may exist in a dormant state with minimal gene expression [80]. Even with ultra-sequencing depth, detecting extremely low abundance microorganisms would be challenging and result in unaffordable costs [79, 81]. We believe that integrating metagenomics and culturomics will provide a more coherent view of microbiome structure and function by encompassing the whole ecosystem structure as well as low-abundance communities.

### Causal mechanism in the gut microbiome

Among human diseases associated with microbes, phenotypes are often linked to only a subset of strains within microbial clades [82]. Access to pure cultures is essential for validating the hypotheses derived from sequencing data. Investigating the causal relationship between the gut microbiome and disease phenotype using in vitro and in vivo models such as germ-free mice or organoids is important for researching, screening, and evaluating bacterial function. The relationship between *Fusobacterium nucleatum* and CRC serves as a notable paradigm [4, 83]. Genomic analysis detected the enrichment of *F. nucleatum*, a common oral anaerobic bacterium, in CRC tumor tissues before isolation for investigating the physiological characteristics and virulence factors [84, 85]. Several hypotheses were proposed and validation experiments followed suit, mounting evidence now shows that tumor-enriched *F. nucleatum* plays a role in multiple stages of CRC progression [85–88]. Clinical applications are currently underway with *F. nucleatum* potentially serving as a diagnostic biomarker, prognostic predictor, and therapeutic target in CRC [83].

Besides, the understanding of the relationship between *Helicobacter pylori* and gastric cancer remains incomplete. Why do only a small percentage of *Helicobacter pylori*-infected individuals develop gastric cancer? Do variations in pathogenicity, virulence, drug resistance, or genetic diversity among *Helicobacter pylori* strains play significant roles in gastric cancer progression? Could other gut microbiota also contribute? These questions are expected to be answered at the strain level through culturomics research. In some studies, metagenomics analysis may yield inconsistent results. Is it due to differences in populations and backgrounds, or the potential confusion caused by host-specific strains? Pure culture research may help clarify this uncertainty. Swift isolation of the target microorganisms could facilitate mechanistic studies in gut microbiome research.

### Biotherapy of the gut microbiome

The clinical application of fecal microbiota transplantation (FMT) has been approved for treating recurrent *Clostridioides difficile* infections (CDI) and severe and fulminant CDI, as stated in guidelines [89]. Currently, FMT is being investigated as a potential treatment for a variety of diseases. Nevertheless, concerns regarding pathogen transmission and the challenge of fully characterizing the composition of fecal samples have restricted its broader application [90]. The exact components responsible for its efficacy or potential negative effects remain unclear, which highlights the importance of obtaining pure cultures from complex communities and fully understanding their interaction mechanism. Ideally, disease- and recipient-specific artificially designed flora should be developed to carry out standardized, and streamlined FMT procedures. The establishment of an extensive strain library through culturomics is the cornerstone of personalized medicine for the gut microbiome.

Probiotics could be a therapeutic approach to modulate the gut microbiota and enhance human health. Traditional probiotics have a well-documented history of use with a proven safety record. In contrast, next-generation probiotics (NGPs) are recently discovered strains whose safety remains unconfirmed. NGPs have been mostly identified through metagenomic analysis before isolation and even modification take place. Research focuses on NGPs such as *Faecalibacterium prausnitzii*, *B. fragilis*, and *Akkermansia muciniphila* [91]. An in-depth understanding of strain level is essential for probiotic research because certain *Akkermansia muciniphila* potentially promote colitis, while enterotoxigenic *B. fragilis* acts as a pathobiont [92, 93]. Utilizing advanced techniques for isolation and identification enables the establishment of a comprehensive strain-level resource library that facilitates the exploration and discovery of novel functional bacteria for the development of probiotics.

## Conclusions

Currently, there is an immediate need for effective methods to isolate, cultivate, and identify gut microbes to establish a comprehensive collection of intestinal strains. The utilization of culture-dependent methods can significantly enhance the precise analysis and thorough exploration of metagenomics by refining reference genomic databases. Acquiring pure cultures is essential for studying disease causation mechanisms and developing personalized microbial therapies. Therefore, it is imperative to revolutionize the methodology used for microbial isolation, cultivation, and identification. Here, we summarize recent progress and challenges in these fields, with a particular focus on the transition from traditional labor-intensive methods to high-throughput and automated approaches. Technological advancements in this domain will facilitate the exploration of the gut ‘dark matter’ within the gut microbiota, enabling comprehensive investigations at the strain level while elucidating mechanisms underlying gut microbiota-related diseases.

## Data Availability

The data and materials used for this review are available within the manuscript.

## Abbreviations

*B. fragilis*: *Bacteroides fragilis*
CDI: *Clostridioides difficile *infections
CRC: Colorectal cancer
FACS: Fluorescence-activated cell sorting
FISH: Fluorescence in-situ hybridization
FMT: Faecal microbiota transplantation
LAMP: Loop-mediated isothermal amplification
MALDI-TOF MS: Mass-assisted laser desorption/ionization time-of-flight mass spectrometry
MB: Molecular beacon
MISS Cell: Single-cell microlitre-droplet screening system
NGPs: Next-generation probiotics
PCR: Polymerase chain reaction
